# Costs of locomotion in polar bears: when do the costs outweigh the benefits of chasing down terrestrial prey?

**DOI:** 10.1093/conphys/cow045

**Published:** 2016-10-15

**Authors:** Linda J. Gormezano, Scott R. McWilliams, David T. Iles, Robert F. Rockwell

**Affiliations:** 1 Division of Vertebrate Zoology, American Museum of Natural History, 79th Street and Central Park West, New York, NY 10024, USA; 2 Department of Natural Resources Science, University of Rhode Island, Kingston, RI 02881, USA; 3 Department of Wildland Resources and the Ecology Center, 5230 Old Main Hill, Utah State University, Logan, UT 84322-5230, USA

**Keywords:** locomotion, polar bear, predation, snow goose, terrestrial prey, *Ursus maritimus*

## Abstract

Trade-offs between locomotory costs and foraging gains are key elements in determining constraints on predator–prey interactions. One intriguing example involves polar bears pursuing snow geese on land. As climate change forces polar bears to spend more time ashore, they may need to expend more energy to obtain land-based food. Given that polar bears are inefficient at terrestrial locomotion, any extra energy expended to pursue prey could negatively impact survival. However, polar bears have been regularly observed engaging in long pursuits of geese and other land animals, and the energetic worth of such behaviour has been repeatedly questioned. We use data-driven energetic models to examine how energy expenditures vary across polar bear mass and speed. For the first time, we show that polar bears in the 125–235 kg size range can profitably pursue geese, especially at slower speeds. We caution, however, that heat build-up may be the ultimate limiting factor in terrestrial chases, especially for larger bears, and this limit would be reached more quickly with warmer environmental temperatures.

## Abstract (precis)

Trade-offs between locomotory costs and foraging gains are key to predator–prey interactions. As climate change forces polar bears to spend more time ashore, their prey base and these trade-offs may be altered. We show that polar bears can profitably capture seasonally available land-based prey, such as geese, over a range of pursuit speeds.

## Introduction

The relationship between energetic gain and locomotory cost is a key determinant in predatory behaviour and greatly influences predator–prey interactions (e.g. Sinclair *et al*., 2003; Scharf *et al*., 2006). In the broadest sense, predatory behaviour of mammalian carnivores spans a range from ambushes [e.g. lions (*Panthera leo*) and leopards (*Panthera pardus*)] to rapid, long-distance pursuits [e.g. cheetah (*Acinonyx jubatus*) and spotted hyena (*Crocuta crocuta*); e.g. Bro-Jørgensen, 2013]. A particularly intriguing case involves the interactions of polar bears (*Ursus maritimus*) and lesser snow geese (*Chen caerulescens caerulescens*), a land-based prey that may become an increasingly important seasonal food resource for polar bears as climate changes (Gormezano and Rockwell, 2013a,b, 2015).

Polar bears normally use the sea ice as a platform to catch marine prey, particularly ringed seals (*Pusa hispida*), and accumulate a majority of their annual fat reserves from consuming seal pups in spring (e.g. Stirling and Øritsland, 1995). In more southern polar bear populations, it is thought that this energy store helps to sustain the bears during the ice-free period each summer (e.g. Stirling and Derocher, 1993; Regehr *et al*., 2007). With warmer temperatures leading to earlier sea ice break-up, access to this energy-rich spring seal diet may become limited, potentially forcing the bears to expend energy seeking land-based food to compensate for energy deficits (e.g. Stirling and Derocher, 2012; Gormezano and Rockwell, 2013a, 2015; Lunn *et al*., 2016). Any increased effort to obtain food is of concern because polar bears are considered inefficient at walking (Øritsland *et al*., 1976; Best 1982; Hurst *et al*., 1982a,b), exhibiting higher rates of oxygen consumption with increased walking speed than predicted for mammals of their size (Taylor *et al*., 1970; Fedak and Seeherman, 1979). The higher rates of energy use have been attributed to their morphology, particularly their large, heavy limbs (Øritsland *et al*., 1976; Hurst *et al*., 1982a,b), a characteristic shared by male lions that likewise have relatively high costs of locomotion (Chassin *et al*., 1976). Despite these energetic limitations, polar bears are known to walk long distances in search of prey on sea ice and land (e.g. Born *et al*., 1997; Amstrup *et al*., 2000; Parks *et al*., 2006; Anderson *et al*., 2008; Rockwell *et al*., 2011) but generally use more energy-conserving stalking or ‘still-hunting’ techniques to capture seals and other marine mammals on the sea ice (e.g. Stirling, 1974; Smith, 1980).

Some polar bears, especially those forced ashore when the sea ice melts in summer, have been observed running on land in pursuit of terrestrial prey (e.g. Brook and Richardson, 2002; Iles *et al*., 2013 and references therein). Given their locomotive inefficiency and potential to overheat in warm weather (Øritsland, 1970; Øritsland and Lavigne, 1976; Best, 1982), it is unclear whether these more intensive pursuits can be energetically profitable (Lunn and Stirling, 1985; Iles *et al*., 2013). In the only examination of this issue thus far, Lunn and Stirling (1985) used a calculation based on Hurst *et al*. (1982a) to suggest that a 320 kg polar bear chasing a goose at 20 km/h for >12 s would expend more energy in the pursuit than could be obtained from consuming it. Despite the speed and mass specificity of that projection, many authors have used this threshold in evaluating observations of polar bears chasing various land-based prey [e.g. caribou, *Rangifer tarandus* (Brook and Richardson, 2002); barnacle geese, *Branta leucopsis* (Stempniewicz, 2006); thick-billed murres, *Uria lomvia* (Donaldson *et al*., 1995); lesser snow geese (Iles *et al*., 2013)] and questioned the energetic worth of the observed predatory behaviours.

The exact energetic costs associated with land-based hunting behaviour are especially important for polar bears in western Hudson Bay, where recent warming trends are rapidly diminishing ice extent and duration (Gagnon and Gough, 2005; Stirling and Parkinson, 2006; Lunn *et al*., 2016). If polar bears come ashore with nutritional deficits (e.g. Stirling and Parkinson, 2006; Regehr *et al*., 2007), any calories obtained on land may become increasingly important for survival (Gormezano and Rockwell, 2013a,b; Gormezano, 2014; Gormezano and Rockwell, 2015) unless the net energetic gain from foods obtained on land exceeds the energetic costs required to obtain them. In western Hudson Bay, snow geese make up an increasing proportion of polar bears’ land-based diet owing in part both to increased temporal overlap of the two species and to greatly increased abundance of snow geese (Gormezano and Rockwell, 2013a, 2015). Given that polar bears in this region spend increasingly more time on land and thus have more opportunities for terrestrial foraging, we constructed predictive models that estimate, for the first time, the metabolic costs of terrestrial locomotion for polar bears of multiple sizes travelling at various speeds. We then use the best-fitting model to evaluate when a polar bear would profit from chasing and catching moulting snow geese, a common terrestrial prey species during summer.

In the following analysis, we revisit the only published data on the metabolic costs of locomotion across a range of speeds for polar bears of multiple sizes. We assess the profitability of pursuing flightless geese using data-driven energetic models that simultaneously account for the effects of polar bear speed and mass. We show that pursuits lasting longer than 20 min in duration can be energetically profitable, although this depends importantly on the speed and mass of polar bears, and that successful pursuits of even distant geese can result in net energetic gains for some polar bears. Furthermore, we show that the smaller-sized and younger bears that could take more advantage of this profitability include those whose survival in western Hudson Bay is lower (Lunn *et al*., 2016) and that may be more impacted by climate change (Regehr *et al*., 2007).

## Materials and methods

To develop a data-driven model that allows oxygen consumption (and thus metabolism) to scale with polar bear speed and mass, we extracted original data from the three published studies that reported measurements of oxygen consumption (${\dot{V}}_{O_{2}}$; in millilitres of O_2_ per gram per hour) as a function of walking speed for polar bears that weighed 125, 155, 190 and 235 kg. The 125 and 155 kg animals were subadult males (as defined by Watts *et al*., 1991), the 190 kg animal was a 4-year-old female (Hurst *et al*., 1982a) and the 235 kg animal was a ~4-year-old male (Øritsland *et al*., 1976). We used the means of the multiple trials of each bear at each speed as the best estimates of O_2_ consumption for each mass and speed. Both linear (Øritsland, 1970; Hurst *et al*., 1982a) and double exponential regression models (Hurst *et al*., 1982a) have previously been used to describe how oxygen consumption changes with speed for bears of different sizes. Here, we first considered three potential models to describe the general shape of the relationship between polar bear speed [*S*; we use this term rather than velocity (*V*) as used by Hurst *et al*., 1982a] and oxygen consumption (${\dot{V}}_{O_{2}}$) using data from Øritsland *et al*. (1976), Hurst *et al*. (1982a) and Watts *et al*. (1991). Our initial model set included the following:

(1) a linear model that allows metabolism to increase at a constant rate with increasing speed,

(1) $${\dot{V}}_{O_{2}}=P+bS;$$

(2) an exponential model that allows metabolism to accelerate with increasing speed,

(2) $${\dot{V}}_{O_{2}}=Pe^{bS};$$

and (3) a double-exponential model that allows metabolism to more flexibly scale with speed,

(3) $${\dot{V}}_{O_{2}}=Pe^{bS^{c}};$$

where *P* is polar bear postural cost (i.e. the energetic cost of maintaining an upright posture when speed is zero), *e* is the natural log (2.718…), and *b* and *c* are exponents that describe the rates at which oxygen consumption changes with movement speed (*S*). From previous work (Hurst *et al*., 1982b), postural costs are known to depend on mass. Thus, in all models we fixed the postural costs at the expected values for each polar bear mass based on the equation of Hurst *et al*. (1982b), following Taylor *et al*. (1970):

(4) $$P=1.056\times {\mathrm{mass}}^{-0.25}.$$

By fixing the postural costs (the *y*-intercept) based on this equation rather than allowing the postural costs to be estimated based on model fit, we improve the biological realism of our models outside the range of our data (i.e. when speed is zero), while only slightly sacrificing goodness of fit within the range of our data (speeds of 1.8–7.92 km/h). We note, however, that results were qualitatively similar whether postural costs were fixed based on Equation 4 or estimated based on our data. We evaluated relative support for the models using Akaike's information criterion (AICc; Akaike, 1973) and found that the exponential and double-exponential models received similar support (Table 1; ΔAICc = 0 and 0.5, respectively), and greatly outperformed the linear model (ΔAICc = 24).

**Table 1:** Model selection results incorporating effects of mass on the relationship between speed and oxygen consumption.

Model	logLik	AICc	ΔLogLik	ΔAICc	parameters	Weight
$Pe^{\mathrm{bS}}$	10.1	−15.5	12	0	2	0.288
$Pe^{bS^{c}}$	11.3	−15	13.2	0.5	3	0.223
$Pe^{\left(b+m1*\mathrm{mass}\right)S^{\left(c+m2*\mathrm{mass}\right)}}$	14.9	−14.7	16.7	0.7	5	0.199
$Pe^{bS^{\left(c+m2*\mathrm{mass}\right)}}$	12.3	−13.6	14.2	1.9	4	0.113
$Pe^{\left(b+m1*\mathrm{mass}\right)S^{c}}$	12.2	−13.4	14.1	2.1	4	0.101
$Pe^{(b+m1*\mathrm{mass})S}$	10.3	−12.8	12.1	2.7	3	0.076
P+bS	−1.8	8.5	0	24	2	<0.001

Model parameters are as follows: *b* and *c*, single and double exponents, respectively; *e*, the natural logarithm (2.718…); *m1* and *m2*, scaling parameters that relate the single exponent and the double exponent, respectively, to polar bear mass; *mass*, polar bear mass (in kilograms); *P*, postural costs; and *S*, polar bear movement speed. In all models, postural costs are described by Equation 4 and thus depend on polar bear mass.

We then constructed several additional models to evaluate potential effects of polar bear mass on oxygen consumption, beyond the effects on postural cost in Equation 4. Given that the exponential and double-exponential models received similar support and produced similar predictions across the range of our data, we constructed a suite of models that allowed mass to influence *b* and/or *c* in Equations 2 and 3 (Table 1). We used AICc and Akaike weights to evaluate relative support among different parameterizations and assess the relative effects of mass and speed on oxygen consumption.

Using model projections of oxygen consumption based on our top model and following Lunn and Stirling (1985), we calculated the time threshold (hereafter, ‘inefficiency threshold’) beyond which the calories expended to chase a goose exceeded the calories obtained from consuming it for polar bears ranging in mass from 125 to 235 kg and over a range of speeds from 0 to 7.9 km/h. For comparative purposes with previous work (Lunn and Stirling, 1985) and because polar bears are known to run at speeds up to 29 km/h (Harrington, 1965), we also projected inefficiency thresholds to 20 km/h. We discuss the assumptions and limitations of those extrapolations in the Discussion.

Estimating the usable energy available to a polar bear eating a goose requires knowledge of (i) the energy in the part(s) of a goose that are eaten, and (ii) the digestibility of the energy in the parts of the goose eaten. Polar bears that successfully capture and eat a variety of prey including seals (Smith, 1980; Best, 1985) and geese (Iles *et al*., 2013, Gormezano and Rockwell, 2015; DTI & RFR personal observations) rarely consume the less digestible portions, including hair and feathers, and usually avoid eating the gastrointestinal tract and the entire skeleton. Thus, we assumed that polar bears primarily consumed the breast, leg muscle, gizzard and fat stores from a captured goose. We estimated the caloric value of these eaten parts of the goose using adult female goose body composition data from Ankney and MacInnes (1978) (as did Lunn and Stirling, 1985) during the post-hatch period, when many instances of predation have been observed (Iles *et al*., 2013). At this post-hatch time, adult female geese (*n* = 35) had negligible amounts of fat and 163.3 ± 4.0 g of protein within the gizzard, breast and leg muscles (Table 3 of Ankney and MacInnes, 1978), which would provide 702.5 kcal, assuming an energy-to-protein conversion of 4.3 kcal/g protein (Robbins, 1993). However, polar bears cannot be expected to digest all the available protein, so some discount is necessary.

Grizzly and black bears digested 89–96% of crude protein in the meat from various mammals and birds (Pritchard and Robbins, 1990), whereas the digestibility of crude protein for bears fed whole birds or mammals was less (85.5 ± 2.2%) because of the non-digestible or less digestible parts (e.g. feathers, hair, skeleton; Pritchard and Robbins, 1990; Robbins, 1993). Likewise, captive polar bears fed various parts of ringed seals (*Phoca hispida*) digested 72–95% of protein nitrogen, with the highest digestibility occurring when polar bears ate seal muscle and viscera and the lowest digestibility when the skeleton, skin and blubber were also eaten (Best, 1985). We assumed that polar bears digested 95% of protein when eating only the gizzard, leg and breast muscle of the goose; digestibility of protein would be much lower (72–85%) if polar bears also ingested other less digestible parts of the whole goose. We present results for the most likely scenario, where polar bears ate the gizzard, leg and breast muscle of the goose and thus gained 667.4 kcal per goose (total of 702.5 kcal, of which 95% was digested).

Finally, to determine the conditions in which inefficiency thresholds would be reached during pursuits of flightless geese, we calculated the duration of pursuits resulting from different combinations of polar bear speeds and initial distances from geese. We assumed that geese fled from pursuing bears at 2 m/s; a value slightly higher (and thus more conservative in terms of polar bear profitability analysis) than the reported maximal sustained running speeds of 0.8–1.2 m/s, considered ‘moderate’ to ‘fast’ for similar sized geese (Codd *et al*., 2005; Hawkes *et al*., 2014). We calculated the time (*t*) required for a polar bear to capture a goose as follows:

(5) $$t=\frac{D}{S_{\mathrm{bear}}-S_{\mathrm{goose}}},$$

where *D* is the initial distance between the bear and the goose, and *S*_bear_ and *S*_goose_ are their respective speeds. For each combination of bear mass, speed and initial distance, we calculated the inefficiency threshold and compared this with the chase duration to determine whether the pursuit resulted in a net surplus of energy for the bear.

All analyses were performed using the R statistical programming language (version 3.2.3; R Development Core Team, 2008).

## Results

**Figure 1:**
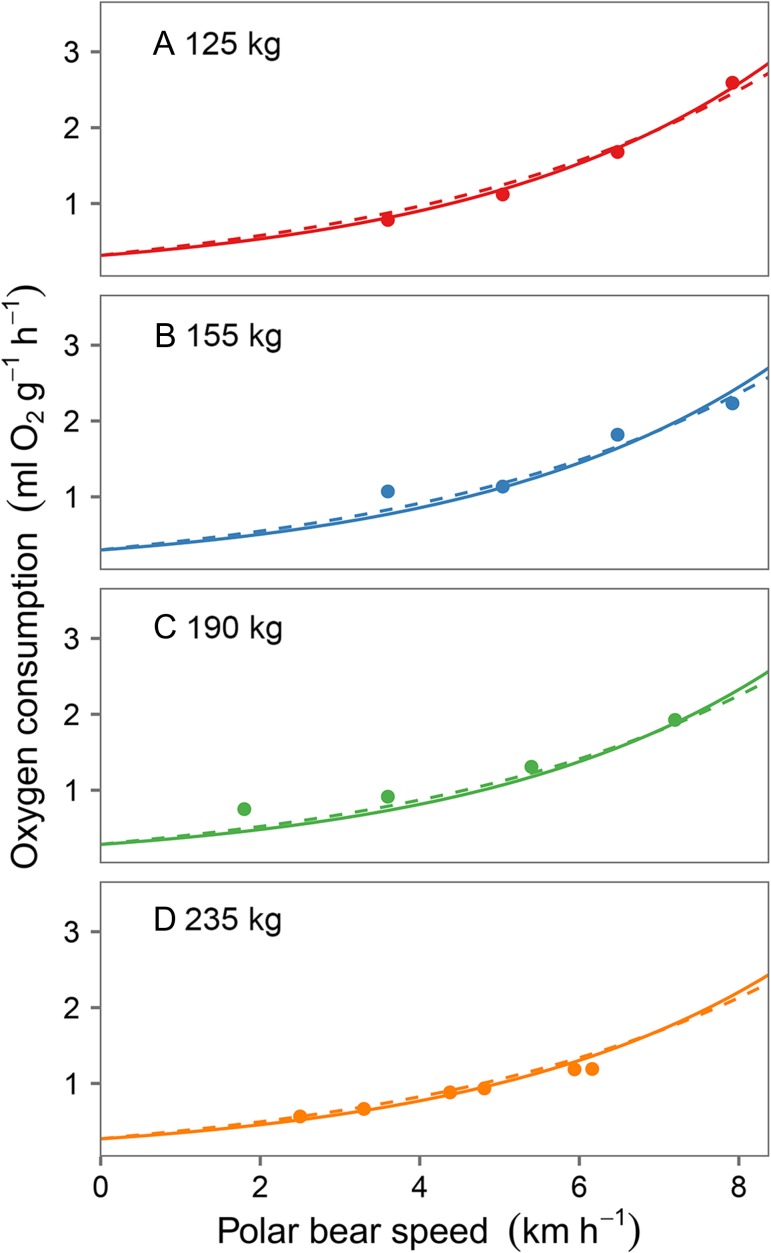
Mass-specific oxygen consumption increases with movement speed. Postural costs (*y*-intercept) are affected by polar bear mass according to Equation 4. The top model based on AICc was a single-exponential model (continuous lines). A double-exponential model received similar support (ΔAICc = 0.5) and made similar predictions across the range of data (dashed lines).

**Figure 2:**
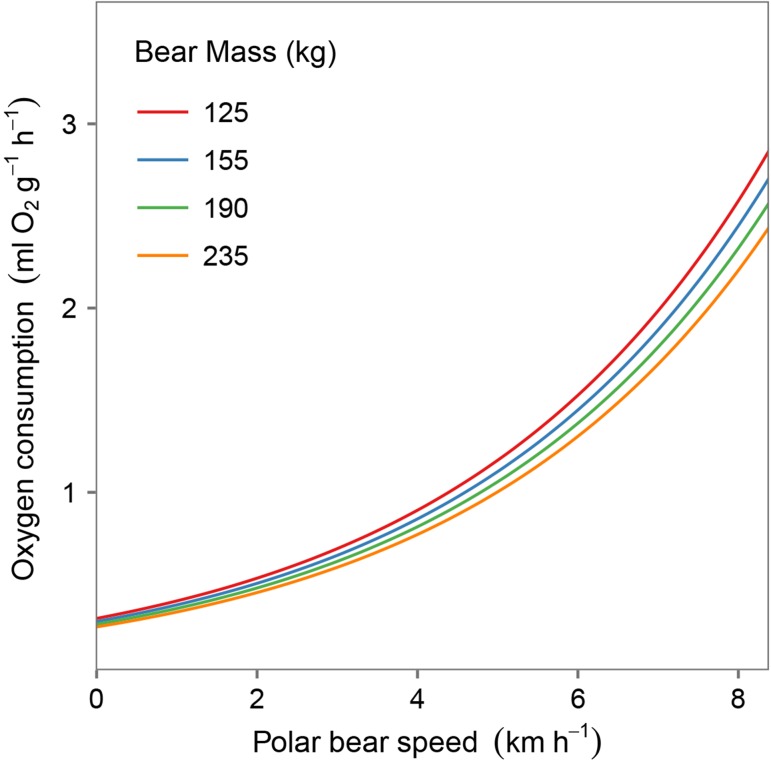
Mass-specific oxygen consumption (${\dot{V}}_{O_{2}}$) increases with movement speed. Postural costs (*y*-intercept) are affected by polar bear mass according to Equation 4. Larger bears are proportionately more efficient than smaller bears. Curves are based on predictions from the top model (exponential model; Equation 2), which when parameterized is: ${\dot{V}}_{O_{2}}$ = (1.056 * mass^−0.25^) * *e*^0.2626*S^.

The relationship between polar bear movement speed and oxygen consumption was best described by either an exponential or a double-exponential model, indicating that metabolism increases exponentially at higher speeds (Fig. 1). We found no support for an effect of polar bear mass on the exponents in either model (Table 1). Given that postural cost depends on polar bear mass (Equation 4) but the shape of the exponential relationship between polar bear speed and oxygen consumption does not, larger bears are more efficient than smaller bears on a proportional basis across all movement speeds (Fig. 2). As the exponential model received slightly higher support and was more parsimonious (i.e. used fewer parameters) than the double-exponential model, we used the exponential model to generate estimates of oxygen consumption as a function of polar bear mass and speed (Fig. 2) and, subsequently, to determine energetic inefficiency thresholds and profitability while chasing flightless geese. We noted, however, that the double-exponential model produced very similar predictions to the top model across the range of data (Fig. 1, compare continuous and dashed lines).

**Figure 3:**
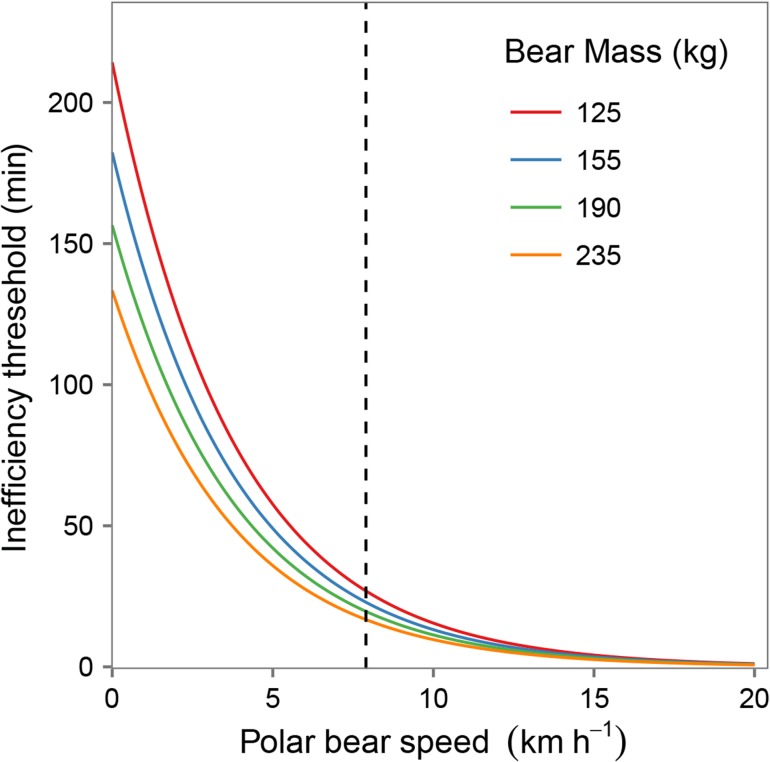
Time ‘inefficiency’ threshold, beyond which the calories expended by a polar bear to chase an adult female goose exceed the calories obtained from consuming it, as a function of speed of the chase and polar bear mass. Note that projections for speeds >7.9 km/h (dashed vertical line) are extrapolations beyond the available data and should be interpreted with caution, but are pictured for comparison with extrapolations by previous studies. The inefficiency threshold (*I*) is calculated as follows: *I* = 667.4/(${\dot{V}}_{O_{2}}$ * mass * 4.735)/60, where 667.4 is the caloric value of a goose, mass-specific oxygen consumption (${\dot{V}}_{O_{2}}$) is estimated as in the legend to Fig. 2, and 4.735 is the standard conversion of 1 litre of oxygen to kilocalories.

Combining results from our oxygen consumption models with the energetic value of a female lesser snow goose, we calculated that a 125 kg polar bear could chase a goose for 26.9 min at 7.9 km/h (the maximal speed of polar bears for which oxygen consumption measurements were recorded) before it becomes energetically unprofitable. In contrast, the inefficiency threshold for a 235 kg bear at 7.9 km/h was 16.7 min. Given that energy consumption increases with speed, the inefficiency threshold decreases with increasing speed for bears of any mass. Despite larger bears having lower proportional oxygen consumption than smaller bears (Fig. 2), the higher absolute mass of larger bears results in lower inefficiency thresholds across the range of speeds for which there are data (Fig. 3). As a consequence, smaller bears can sustain chases that are longer in duration.

**Figure 4:**
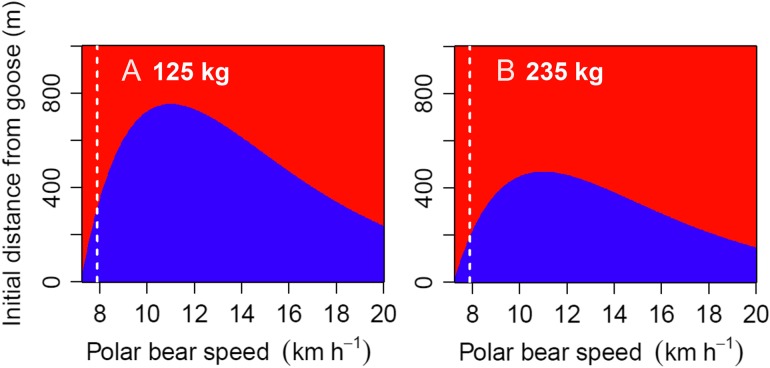
Profitability of capturing flightless snow geese for polar bears weighing 125 (**A**) or 235 kg (**B**). The initial distance from flightless geese and polar bear speed influence the time required to capture a goose, whereas polar bear mass and speed influence the inefficiency threshold (chase duration beyond which energy expenditures exceed energy gains from consuming a 667.4 kcal goose). Chases that are shorter in duration than the inefficiency threshold are coloured in blue (resulting in a net energy surplus for polar bears). Note that because geese are capable of running at 2 m/s (or 7.2 km/h), bears are incapable of capturing geese when moving slower than this speed. Areas to the right of the white dashed lines are extrapolations outside the range of data, but are pictured for comparison with extrapolations in previous studies.

Ultimately, the time required to capture terrestrial prey depends on the initial distance between the polar bear and prey and the relative speeds of the bear and the prey. If the chase duration exceeds the energy inefficiency threshold for that particular pursuit speed, polar bears will lose energy even from pursuits in which they successfully capture geese. We found that polar bears were capable of capturing geese before reaching their inefficiency threshold for a wide range of pursuit scenarios (Fig. 4, blue areas). Smaller bears (i.e. 125 kg) were capable of gaining energy from pursuits of geese up to 754 m away, whereas larger bears (i.e. 235 kg) could gain energy from pursuits of geese up to 468 m away.

## Discussion

The best-supported predictive model for estimating the metabolic costs of terrestrial locomotion for polar bears of different sizes was a simple exponential model (Fig. 2). Importantly, the shape of the exponential relationship between polar bear speed and metabolic cost did not depend on polar bear mass, and only the postural costs (*y*-intercept) were mass dependent; the implication being that smaller bears therefore spend proportionately more energy for locomotion than larger bears (Fig. 3). Previous studies have shown that postural costs (energy costs when speed is zero) are greater for smaller bears (Scholander *et al*., 1950; Hurst *et al*., 1982b), a pattern observed in smaller and immature animals in general (Taylor *et al*., 1970; Lavigne *et al*., 1986). These higher postural costs with decreasing polar bear mass combined with similar exponential increases in the energy costs of locomotion with travel speed regardless of mass result in smaller bears having proportionately higher locomotion costs than larger bears at a given travel speed.

Earlier studies have suggested that the higher locomotive costs of smaller bears could be related to increased stride frequency, because more steps will be needed to maintain the same speed as larger bears (Heglund and Taylor, 1988; Best *et al*., 1981). Energy cost per gram of body weight per stride is relatively constant across animals of drastically different masses moving at the same speed (Heglund *et al*., 1982), so although heavier animals require more energy to move per stride, the longer stride length and lower stride frequency could result in increased efficiency over the same distance (Heglund *et al*., 1982). Incremental rates of energy use during terrestrial locomotion can also change with transitions to different gaits (Chassin *et al*., 1976; Heglund and Taylor, 1988; Reilly *et al*., 2007; Watson *et al*., 2011), although this has not yet been studied in polar bears and warrants further attention because it could affect the shape of oxygen consumption curves at higher speeds.

Pursuits (and capture) of flightless snow geese lasting longer than 12 s have been documented (Iles *et al*., 2013), and we have observed multiple examples of this behaviour in recent years (LJG & RFR our unpublished data). Our analyses here indicate that these observations are to be expected, given that prolonged (i.e. >20 min) pursuits of even distant geese (i.e. farther than 500 m) can be energetically profitable, especially for polar bears in the size range for which there are data (Figs 3 and 4). Of those, smaller bears are capable of profitably engaging in pursuits of more distant geese and at higher pursuit speeds, given their lower overall level of energy expenditure (Fig. 4). In western Hudson Bay, subadult polar bears (those that are included in the studied size range) as well as females with cubs tend to arrive onshore in spring earlier than larger, mature individuals (Rockwell and Gormezano, 2009). Interestingly, our results suggest that these younger and smaller bears, which have recently been shown to have lower survival (Lunn *et al*., 2016) and which may be disproportionately affected by lost opportunities to hunt seals as a result of climate change (Regehr *et al*., 2007; Rockwell and Gormezano, 2009), should have an inherently better ability to recover caloric deficits via terrestrial prey.

Prolonged chases of flightless snow geese can be energetically profitable over a range of pursuit speeds for polar bears in the 125–235 kg size range. The same is likely to be true for larger bears, those outside the range of available oxygen consumption data, because only postural cost (*y*-intercept) is mass dependent and it scales at the 0.25 power (Fig. 4; Taylor *et al*., 1970). Extrapolations past the upper limit of speeds for which there are data assume that the functional basis for the modelled trend remains the same, an assumption that may be violated if polar bears change gait and energy efficiency at higher speeds. Nevertheless, based on our top model, we project that a 320 kg bear running at 20 km/h would expend the calories contained in an adult goose in 33 s, a value that is reasonably comparable to the estimate of 12 s previously suggested by Lunn and Stirling (1985) using a different model. However, we note that our model also predicts that 320 kg bears can more profitably engage in much longer pursuits at slower speeds (e.g. our model predicts that pursuits of geese lasting up to 13.3 min are energetically profitable for a 320 kg polar bear running at 7.9 km/h).

Although polar bear locomotion is considered relatively inefficient, they typically walk slowly, with a steady gait of ~5.5 km/h (Stirling, 1988). They average 1–5 km/h over longer distances, periodically interspersed with rest stops, and can sustain these speeds for extended periods while covering large distances (Harrington, 1965; Amstrup *et al*., 2000; Anderson *et al*., 2008; Durner *et al*., 2011; Whiteman *et al*., 2015). For example, Amstrup *et al*. (2000) reported many polar bears sustaining average travel on the ice at >4 km/h for up to 20 h, with some maintaining these speeds for >40 h. In a controlled experiment, polar bears trained to walk on treadmills were likewise able to walk for long periods, continuing exercise for up to 90% of 6 h walking sessions (Best, 1982). However, during these trials the polar bears thermoregulated behaviourally by leaving the treadmill temporarily to ingest snow when their core temperatures reached a particular threshold (Best, 1982). Best (1982) suggested that hyperthermia, not fatigue, was more likely to be a limiting factor to continuous locomotion. Polar bears have also been observed sustaining higher speeds (approaching 10 km/h) for shorter periods of time while on the ice (i.e. 1–8 h; Amstrup *et al*., 2000), where low ambient temperatures and strong winds would be likely to reduce the risk of hyperthermia (Best, 1982).

In contrast, while on land during the ice-free season in western Hudson Bay, when ambient temperatures are considerably higher, polar bears limit their daily movements, remaining inactive for long periods (Knudson, 1978; Latour, 1981). However, they have been observed engaging in faster-paced pursuits after caribou and waterfowl (e.g. Brook and Richardson, 2002; Iles *et al*., 2013; LJG & RFR our unpublished data). In such cases, hyperthermia, rather than lack of profitability, may be a limiting factor to sustained activity for several reasons. Polar bears are typical of non-sprinting mammals in that almost all the heat produced during exercise is immediately dissipated and little is stored (Taylor *et al*., 1970; Best, 1982), making warmer ambient temperature conditions particularly problematic because they reduce the potential for heat dissipation during exercise. For example, 218–239 kg polar bears walking at 7.9 km/h reached their upper critical temperature (when core body temperature can no longer be regulated) at about −33°C. Furthermore, these captive bears could sustain this activity at temperatures only up to −20°C when allowed to ingest snow before returning to walk (Best, 1982).

**Figure 5:**
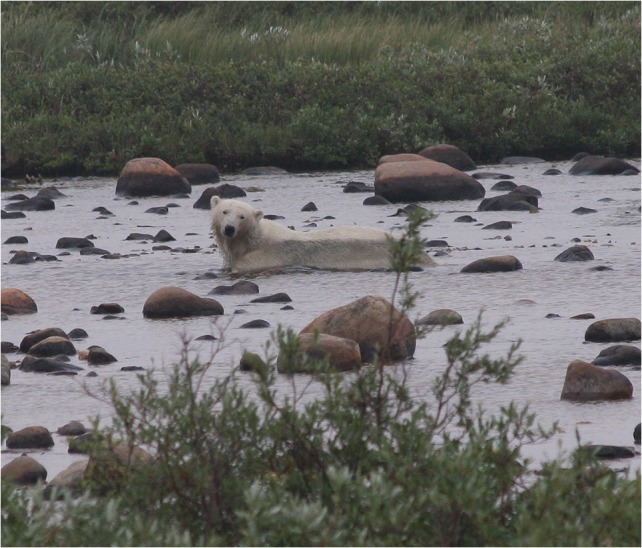
A subadult male polar bear in the Mast River (Wapusk National Park) after killing at least five flightless snow geese in three chases. After the chases, the bear walked into the river, lay down and drank periodically. Photographed on 13 July 2013 by R.F.R.

Interestingly, many pursuits by wild bears have been observed in or near ponds, lakes and rivers (Iles *et al*., 2013; LJG & RFR our unpublished data), with the bear often lying in shallow streams and ponds immediately after the pursuit (Fig. 5). Immersion in water has been shown to reduce a polar bear's core body temperature substantially both before and after sustained exercise (Øritsland, 1969; Frisch *et al*., 1974). In general, the thermoregulatory costs of exercise for polar bears can be somewhat dissipated by certain behaviours, but these costs probably often constrain the duration and speed of a wild goose chase, especially during warm summer days.

Additional research is clearly needed to gain a full understanding of the thresholds of inefficiency of foraging pursuits associated with polar bear locomotion. This is especially true for larger-sized bears and for all bears travelling near their maximal speeds. Such data are crucial for understanding the potential importance of land-based foraging behaviour. Polar bears currently consume various foods on land (e.g. Gormezano and Rockwell, 2013a,b and references therein), but the profitability of these foods and their contribution towards the persistence of polar bears in the face of climate change remains debatable (e.g. Gormezano and Rockwell, 2015; Rode *et al*., 2015; Pilfold *et al*., 2016). To clarify these issues, studies are required either that provide complete data allowing the calculation of energetic and nutritional costs and gains or (preferably) that allow those costs and gains to be measured directly.
